# Spectrotemporal Response Properties of Core Auditory Cortex Neurons in Awake Monkey

**DOI:** 10.1371/journal.pone.0116118

**Published:** 2015-02-13

**Authors:** Roohollah Massoudi, Marc M. Van Wanrooij, Huib Versnel, A. John Van Opstal

**Affiliations:** 1 Department of Biophysics, Donders Institute for Brain, Cognition and Behavior, Radboud University Nijmegen, Heyendaalseweg 135, 6525 AJ Nijmegen, The Netherlands; 2 Department of Otorhinolaryngology, Donders Institute for Brain, Cognition and Behavior, Radboud University Medical Centre Nijmegen, P.O. Box 9101 HB Nijmegen, The Netherlands; 3 Department of Otorhinolaryngology and Head & Neck Surgery, Brain Center Rudolf Magnus, University Medical Center Utrecht, P.O. Box 85500, 3508 GA Utrecht, The Netherlands; University of Salamanca- Institute for Neuroscience of Castille and Leon and Medical School, SPAIN

## Abstract

So far, most studies of core auditory cortex (AC) have characterized the spectral and temporal tuning properties of cells in non-awake, anesthetized preparations. As experiments in awake animals are scarce, we here used dynamic spectral-temporal broadband ripples to study the properties of the spectrotemporal receptive fields (STRFs) of AC cells in awake monkeys. We show that AC neurons were typically most sensitive to low ripple densities (spectral) and low velocities (temporal), and that most cells were not selective for a particular spectrotemporal sweep direction. A substantial proportion of neurons preferred amplitude-modulated sounds (at zero ripple density) to dynamic ripples (at non-zero densities). The vast majority (>93%) of modulation transfer functions were separable with respect to spectral and temporal modulations, indicating that time and spectrum are independently processed in AC neurons. We also analyzed the linear predictability of AC responses to natural vocalizations on the basis of the STRF. We discuss our findings in the light of results obtained from the monkey midbrain inferior colliculus by comparing the spectrotemporal tuning properties and linear predictability of these two important auditory stages.

## Introduction

Many acoustic properties, such as sound spectrum, sound duration, and sound level, but also perceptual qualities, like pitch or binaural disparities, are processed at subcortical levels [1]. The core auditory cortex (AC) is the first stage receiving this preprocessed acoustic information. To study the presumed role of AC in higher-order auditory processing thus requires the use of sounds that contain other, more complex, properties that vary in both the spectral and temporal domains. Useful naturalistic stimuli that meet these requirements are so-called dynamic ripples [2–6]. Although ripples lack the higher-order statistical properties of natural sounds [6] and the 1/f dynamics [7] that cortical neurons might be sensitive to, they are particularly useful to quantify and compare neural tuning characteristics, because they can be described and varied in a straightforward, parametric way.

Ripples share many properties with natural sounds, and they provide adequate information to extract a neuron’s spectrotemporal receptive field (STRF). The STRF is a linear representation of the joint temporal and spectral sensitivity of an auditory neuron [8, 9]. An interesting question is whether a neuron treats time and spectrum as independent variables. If so, the STRF is separable, because it can be decomposed into the product of a spectral and a temporal sensitivity function. In contrast, when a neuron’s STRF is inseparable, it is most sensitive to a particular combined change in time and frequency (like in an upward or downward FM sweep). While recordings in cat and ferret AC have indicated inseparable STRFs in a significant proportion of neurons, most AC cells have separable STRFs [4, 6]. Also in the midbrain inferior colliculus (IC) of the monkey, the majority of cells have separable STRFs, with about 30% of the neurons being inseparable [10].

As the STRF is a linear response kernel of the cell under study, it can also be used to make a linear prediction of the cell’s responses to arbitrary sounds. Versnel et al. [10] reported a considerable linear predictability of the neural responses from a large fraction of IC neurons recorded in alert monkeys. Although studies have also reported linear properties for AC neurons [3, 11, 12], other reports have demonstrated non-linear characteristics [6, 13–15]. For example, Atencio and Schreiner [6] demonstrated that so-called fast-spiking units are more separable and more linear than regular units. On the other hand, they did not obtain significant differences in separability or linearity between neurons with either broad or narrow frequency tunings [15]. Note that most studies have been performed in anesthetized animal models. Anesthesia, however, may affect crucial response properties, such as inhibitory mechanisms [16], which could in turn affect spectral-temporal separability and linearity of the cells [17], and their sustained responsiveness [18].

Studies of AC cells in awake monkey have so far focused on different aspects of neural responses, often in the context of complex sounds, or perception and overt behavior, rather than on basic acoustic spectral-temporal response properties (see, however, [3] which addressed linearity using STRFs, and [19] which distinguished cells based on basic onset and offset responses). For example, AC cells can be modulated by non-acoustic signals [20–26], by complex sounds like conspecific vocalizations [27–29], or by pitch [30, 31]. However, the properties of STRFs and linearity of AC cells in awake monkey have so far not been studied in detail.

We therefore examined the STRFs of AC cells from two awake monkeys using dynamic ripples, and quantified selectivity to ripple density, ripple velocity and direction, as well as their spectral-temporal separability. By comparing the best frequencies for tone responses to those of ripple responses, and the predictability of responses to a set of natural stimuli, we also assessed a neuron’s response linearity. By comparing our earlier results from monkey IC [10] with the current AC recordings, we aimed to clarify the IC-to-AC spectrotemporal processing of sound.

## Materials and Methods

### Subjects

We performed single-unit recordings in the left auditory cortex of two adult male rhesus monkeys (*Macaca mulatta*, Monkey J; 7–9 kg, and monkey T; 8–10 kg). Part of the data acquired from these recordings was presented in other papers [25, 26].

### Ethics Statement

Monkeys were obtained from the national primate research center (BPRC) in Rijswijk, The Netherlands. Animals were housed in the Central Animal Facility (CDL) of the Radboud University, and participated in the recording sessions for about two years. Experiments were conducted in accordance with the European Communities Parliament and Council Directive (September 22, 2010, 2010/63/EU). All experimental protocols were approved by the local Ethics Committee on Animal Research of the Radboud University Nijmegen (RU-DEC, ‘Radboud University Dier Experimenten Commissie’).

Monkeys were pair-housed to facilitate normal interactive behavior, including grooming. Their joined cages measured 1.6 × 2.4 × 2.0 m (height × width × depth), and cage enrichment was provided in the form of a swing, plastic 3D puzzles, a mirror, and tools. The room, in which four such paired cages were placed, was further enriched with soft background pop-music from a Dutch radio station (Sky Radio; 9–16 hours on almost every week day, provided by the Animal Care Facility). To promote foraging behavior, small seeds were dispersed across the floor bed on a daily basis. All animals in the room received a fixed amount (300 g) of dry food daily, and when outside the experimental sessions they each had daily access to a bottle containing 400 ml of water.

About 24 hours before the start of an experimental session, water intake of the monkey was limited to 20 ml/kg. In the experiment, the animal earned a small water reward of 0.2 ml per successful trial. We ensured that the animals earned at least the minimum of 20 ml/kg of water on an experimental day, by supplementing after an experimental session, if needed. Additionally, the animals received fruit. In weekends, the animals’ fluid intake was increased to 400 ml daily.

To monitor the animal’s health status, we kept records of body weight, and water and food intake. Expert veterinarian assistance was available on site. The facility’s expert animal technician performed the surgeries on the animals, described below. Quarterly testing of hematocrit values ensured that the animal’s kidney function remained within the normal physiological range. Our procedures followed the water-restriction protocol of the Animal Use and Care Administrative Advisory Committee of the University of California at Davis (UC Davis, AUCAAC, 2001). Whenever an animal showed signs of discomfort, or illness, experiments were stopped and the animal was treated until the problem was solved.

The animals were sacrificed at the end of the study by an intravenous injection of 1 ml of heparin, followed by an overdose of pentobarbital. The animals were then perfused, and their brains were removed.

### Surgical procedures

After completing training in order to respond to spectrotemporal ripple stimuli at a performance level of at least 80% (see details in [25, 26]), the animal underwent surgery under full anesthesia and sterile conditions. Anesthesia was maintained by artificial respiration (0.5% isoflurane and N2O), and additional pentobarbital (IV; 3 mg/kg/hour), ketamine (IM; 0.1 ml/kg), and fentanyl (IV; 20 μg/kg/hour) were administered. A stainless steel recording chamber (12 mm diameter) was placed over a trepanned hole in the skull (10 mm diameter). The orientation and coordinates of the chamber were directed to the auditory cortex, as determined on the basis of MRI images. The chamber allowed a vertical approach of the left AC. A stainless-steel bolt, embedded in dental cement on the skull, allowed firm fixation of the head during recording sessions.

### Experimental setup

The head-restrained monkey sat in a primate chair within a completely dark and sound-attenuated room (2.45 × 2.45 × 3.5 m), while a glass coated tungsten microelectrode (impedance 1–2 MΩ; Alpha Omega, Ubstadt-Weiher, Germany) was carefully positioned and lowered into the brain through a stainless steel guide tube by an electronically-driven stepping motor (National Aperture Inc. MM-3M-F-1). Electrode signals were grounded to a contact mounted in the skull. The analog electrode signal was amplified (BAK Electronics; model A-1), band-pass filtered between 0.1 and 12.5 kHz (custom-built 8^th^ order LP Butterworth filter in series with Krohn-Hite, model 3343, 100 Hz HP cut-off), and monitored through a speaker and oscilloscope. The raw signal was then digitized (at 25 kHz, A/D convertor, TDT2 system; module AD-1; Tucker-Davis Technologies). An automated algorithm that was controlled by the BrainWare program (V 9.07 for TDT, under Windows 98; DELL PC) detected individual action potentials. Data analysis and spike sorting was performed offline in MATLAB (version 7.14.0.739, R2012a, Natick, MA, USA).

### Sound stimuli

Sound stimuli were digitally generated at a sampling rate of 100 kHz and delivered via the BrainWare software package and TDT2 hardware. A trigger, provided by a TG6 module, started sound presentation (DA1, low-pass filtered at 20 kHz through a TDT2-FT6 module), and spike data acquisition. A speaker (Blaupunkt PCxg352, flat frequency characteristics within 5 dB between 0.2 and 20 kHz), positioned at the frontal central position at a distance of 1.0 m from the monkey, presented the stimuli; sound levels were set by two programmable attenuators (PA4).

The ambient background acoustic noise level was about 30 dBA. Acoustic foam that was mounted on the walls, floor, ceiling, and every large object in the room effectively absorbed reflections above 500 Hz. In this study, we presented three types of acoustic stimuli: (1) pure tones, and (2) frozen static noise, followed by a spectrotemporal ripple, and (3) vocalizations.

**Tones.** Pure tones lasted for 150 ms and were presented over a frequency range from 250 to 16000 Hz at 4 different sound levels [10, 30, 50, and 70 dB sound pressure level (SPL)]. Trials were presented at least four times in a randomized manner. The frequency-tuning curve of a neuron was determined from the average firing rate over 50 ms of onset response for each tone across all sound level presentations. The best frequency (BF_tone_) of each neuron was taken at the maximum of the tuning curve. The cell’s response onset latency was defined as the moment after the pure tone onset at which the mean firing rate across all tones exceeded the mean baseline activity plus twice its standard deviation (SD) for the first time for at least 10 milliseconds.

**Figure 1:**
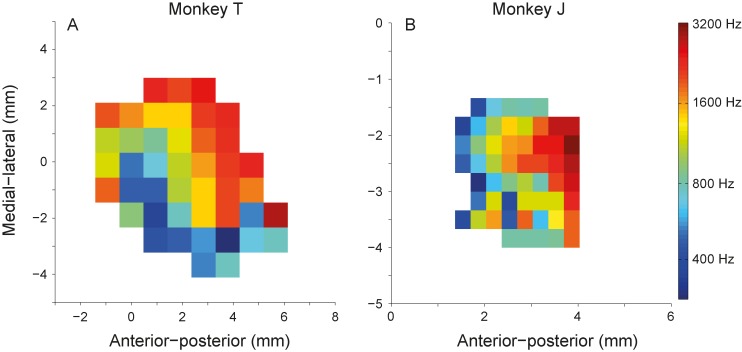
Reconstructed tonotopic map for monkey T (A) and monkey J (B). Each bin represents the average characteristic frequency obtained at the recording location. (A) Characteristic frequency (color coded) increases systematically from anterior to posterior recording sites, and along the medial-lateral direction, indicative for primary auditory cortex (A1) and area R. (B) Characteristic frequency increases only in anterior-posterior direction implying the recorded site were most likely from A1.

For the purpose of reconstructing a tonotopic map (Fig. 1), we also determined the characteristic frequency (CF), as follows. First, the spontaneous firing rate was determined during a 300 ms pre-stimulus period across all (≥ 208) tone stimulus presentations. Second, driven responses were defined as the average firing rate of all (≥ 4) presentations of each tone stimulus that was greater than the mean plus two standard deviations of the spontaneous activity. Finally, the CF of each neuron was defined as the frequency that produced a driven response at the lowest intensity (threshold).

**Ripples.** The ripple stimuli consisted of a broadband complex of 126 spectral components, equally distributed (20/octave) from 250 Hz to 19.7 kHz [4, 10]. All components had random phase. The ripple envelopes were sinusoidally modulated in the spectrotemporal domain. The amplitude of each component was described as follows:

S(t,x)=1+sin(2πωt+2πΩx) ( Eq. 1 )

with t time (in s), x position of the spectral component in octaves above the lowest frequency (250 Hz), ω ripple velocity (Hz; temporal modulation), Ω ripple density (cycles/octave, or c/o; spectral modulation). The set of 55 different ripples used in our study consisted of all combinations of 11 different ripple densities, Ω in [−2.0: 0.4: +2.0] c/o, and 5 different velocities, ω in [8: 8: 40] Hz. A negative density corresponds to an upward direction of the spectral envelopes, a positive density to a downward direction, and Ω = 0 c/o means a pure amplitude-modulated (AM) spectrally flat complex [10]. The ripples with Ω ≠ 0 c/o are referred to as moving ripples, and the ripples with Ω = 0 c/o as AM complexes. For all ripples the modulation depth was 100%. The sound level was 60 dB SPL, and the duration was 1000 ms.

**Vocalizations.** Three different macaque calls and three different bird calls were presented to both monkeys. The bird calls and macaque vocalizations are described in detail in Versnel et al. [10]. Briefly, all vocalizations had been resampled to 50 kHz. Artifacts and background noises were removed by cutting and high-pass filtering. The calls were presented at three sound levels (40, 50, and 60 dB SPL); their durations varied from 300 to 1600 ms, and the number of repetitions in each recording was at least 10.

### Experimental paradigms

Neural responses were measured while monkeys were exposed to the spectral-temporal ripples. The data presented here were collected during recording sessions in which also the AC responses were recorded during a behavioral task (see [25, 26]). Each stimulus started with a static epoch (ω = 0 Hz, Ω = 0 c/o, 500 ms duration, presented at 60 dB SPL), which changed into a pseudo-randomly selected ripple that lasted for 1000 ms (Eq. 1). The static noise was frozen within each block of trials.

Each trial started automatically with the static noise. Data collection started 300 ms before sound onset, and ended 700 ms after sound offset, yielding a total recording duration of 2500 ms. The number of trial repetitions was between 4 and 10.

### Characterization of recording sites

Although we cannot with certainty identify the exact AC subdivision(s) in which we encountered individual neurons, we are confident that we recorded from the AC core (primary auditory cortex A1 and its immediate rostral part, area R) for the following reasons: 1) MRI scans were used for stereotaxic placement of the recording chamber; the subsequent coordinates of the successful recording sites within the chamber corresponded closely to the stereotaxic coordinates of A1 as provided by the atlas of the Rhesus monkey brain [32]; 2) before reaching an AC recording site there was a physiologically silent period, corresponding to the gap between upper and lower parts of the lateral sulcus [33]; 3) tone-onset latency of the recorded sites was 22.6 ± 5.9 and 23.6 ± 5.6ms for monkey J and T, respectively; 4) almost all neurons (96%) responded well to pure tones (BF: 250–16000 Hz); 5) The pure-tone tuning bandwidths for monkey J and T were 1.5 ± 1.2 and 1.5 ± 1.3 octaves, respectively; 6) The pure-tone thresholds for monkey J was 21 ± 13 dB SPL, and for monkey T was 23 ± 12 dB SPL. These tuning characteristics all fall in the same ranges as reported by Recanzone et al. [34] for behaving monkeys in AC areas A1 and R [25, 26]. 7) Finally, we reconstructed tonotopic maps of the recording sites in both animals (Fig. 1). The maps demonstrated a systematic increase in CF from anterior to posterior locations over a spatial extent that corresponded well to earlier studies (e.g., [24]). The tonotopic map obtained for monkey T (Fig. 1A) shows first a decrease in CF, followed by an increase along the anterior-posterior axis, indicative for an area between R and A1. The tonotopic map for monkey J (Fig. 1B) showed a general increase in CF along the anterior-posterior axis, indicating that recordings in this animal were most likely taken from A1.

Our database consisted of 426 cells from which we successfully recorded during presentation of the entire set of 55 ripples (monkey J: n = 178; monkey T: n = 248).

### Data analysis

**The spectrotemporal receptive field (STRF).** We estimated a cell’s STRF from the neural responses to ripple stimuli by using the same off-line method as described before [4, 10]. First, detected spikes were sorted and binned into peri-stimulus time histograms. As stated above, the ripple stimuli followed the static epoch. This caused the ripple onset response to be dramatically suppressed, or fully eliminated [25, 26]. However, to avoid any conflict with potential transient onset responses, we excluded the first 100 ms of the ripple-response window [2, 4, 11]. We then wrapped the 900-ms response window into 32-bin period histograms, in which the ripple velocity determined the period as 1/ω. We subsequently performed a fast Fourier transformation on the period histograms. The magnitude *A(ω, Ω)* (spikes/s) and phase *Φ(ω,Ω)* (rad) of the period histograms were derived from the first harmonic of the resulting Fourier spectrum to generate the modulation transfer function:

T(ω,Ω)=A(ω,Ω)exp(iφ(ω,Ω))
( Eq. 2 )

The 2D inverse Fourier transformation of *T(ω,Ω)* then produces the spectrotemporal response field, or STRF, of the cell:

$$STRF(t,x)=FFT^{-1}[T(\omega ,\Omega )]$$ ( Eq. 3 )

with *x* the frequency in octaves (between 0 and 2.5 in 0.25 octave steps), and *t* running from 0 to 125 ms (at 12.5 ms resolution). The spectral dimension (abscissa) of the STRF reflects the frequency tuning, and the temporal direction (ordinate) reflects the cell’s linear impulse response. The frequency range of the STRF is determined by the step size of the ripple densities employed in the experiment: [range x] = 1/ [step size Ω] 1/[0.4] = 2.5 octaves. Likewise, the temporal range is determined by the resolution in applied ripple velocities: [range t] = 1/[step size ω], leading to 125 ms. The position of the frequency range (in Hz) is ambiguous, as the lower frequency of the STRF could be either 250 Hz (the lowest component in the ripple stimuli), or multiples of 2.5 octaves above 250 Hz (i.e., at 1414 or 8000 Hz). The pure-tone responses were used to resolve this ambiguity.

**The encoding of ripple amplitude modulations.** To quantify how well a cell followed the modulation period of a particular ripple stimulus, a measure, q, was computed as follows [10]:

$$q=\frac{A_{1}}{\sqrt{\sum_{i=1}^{16}A_{i}^{2}}}$$ ( Eq. 4 )

with A_i_ the amplitude of the i-th harmonic of the ripple’s period (1/*ω*). The parameter q reflects the quality for the best sinusoidal fit with the period histogram. If q = 1 the period histogram corresponds to a single period of a pure sinusoid, and suggests a linear response of the neuron. If q = 0 the period histogram does not resemble a single-period sinusoid at all.

We applied q as an index to judge the sensitivity of the neuron to the ripple stimulus. Specifically, we determined the 25^th^ percentile of the q-values, q_25_, for upward (Ω < 0) and downward (Ω > 0) moving ripples (25 ripples each) and the 50^th^ percentile (median), q_50_, for AM complexes (Ω = 0, 5 ripples) for every neuron. Then, through simulation of random period histograms, we determined that 99% of the time a q_25_ value less than 0.387 is found by chance. Similarly, for the q_50_ value a 99% level of 0.376 is observed. Thus, for instance, it is unlikely (p < 0.01) that q_25_ > 0.387 if a neuron responds in a random way to moving ripples.

**Best ripple density and velocity and direction selectivity.** Three response parameters were derived from the transfer function T (Eq. 2): the best ripple velocity ω_B_ of a cell, its best ripple density Ω_B_, and its direction selectivity D. The parameters ω_B_ and Ω_B_ were determined as the Ω and ω at which the magnitude transfer function was the largest. We computed the direction selectivity, D[35]:

$$D=\frac{R_{up}-R_{down}}{R_{up}+R_{down}}$$ ( Eq. 5 )

with R_up_ and R_down_ the sum of the response magnitudes to all upward (Ω < 0) and downward (Ω > 0) ripples, respectively. We also determined the moving ripple/AM response magnitude ratio to indicate the preference of a neuron for moving ripples versus AM complexes. To that end, the magnitude values to the best Ω ≠ 0 were summed for all presented velocities and divided by the sum of the responses to Ω = 0 at all velocities.

$$\frac{bestmovingripple}{AM}=\frac{\sum M_{\Omega_{\text{B}}\neq 0}}{\sum M_{\Omega =0}}$$
( Eq. 6 )

Finally, a best frequency (BF_strf_) and latency were derived as the frequency and latency at which the STRF had its maximum response.

**Separability.** We examined whether the two-dimensional modulation transfer function *T*(*ω*, Ω) can be obtained by the product of two separate, one-dimensional, transfer functions, namely a temporal function *F*(*ω*) and a spectral function *G*(Ω). We applied two different methods to examine the extent of this so-called separability.

First, we performed singular value decomposition (SVD) of the modulation transfer function [4]. For a fully separable function, the following equation applies:

T(ω,Ω) = F(ω)×G(Ω)
( Eq. 7 )

Briefly, the SVD method decomposes *T*(*ω*, Ω) as follows: *T*(*ω*, Ω) = *F*(*ω*) × S × *G*(Ω), with *S* a diagonal matrix with eigenvalues λ_i_ with λ_1_ ≥ λ_2_ ≥ λ_3_ and so forth. For a separable function, λ_1_ = 1, and all other eigenvalues are zero. As a measure to quantify separability, we defined the inseparability index, α, as used by others [4, 5, 10]:

$$a=1-\frac{\lambda_{1}^{2}}{\sum_{i=1}^{n}\lambda_{i}^{2}}$$ ( Eq. 8 )

with λ_*i*_ the eigenvalues. The transfer function is inseparable when α = 1 and completely separable for α = 0.

Second, a temporal transfer function, *F_B_*(*ω*), at Ω = Ω_B_, and a spectral function, *G_B_*(Ω), at *ω* = *ω_B_* were selected from the recorded complete transfer function *T*(*ω*, Ω). We then computed an estimate *T*_est_(*ω*, Ω) according to Eq. 7, *T*_est_(*ω*, Ω) = *F_B_*(*ω*) × *G_B_*(Ω), and correlated the resulting STRF_est_ (Eq. 3) with the original STRF. The correlation coefficient, ρ, was used as an additional measure of separability, with ρ = 1 indicating complete separability and ρ = 0 indicating inseparability

The two separability analyses were performed for the complete transfer function (−2 ≤ Ω ≤ 2 c/o), as well as for each of the two quadrants of the transfer function (Ω < 0 and Ω > 0). The former measure quantifies full separability, whereas the latter quantifies the so-called quadrant separability [4, 10].

**Phase functions.** For a majority of transfer functions, the phase functions Φ(*ω*, Ω) appeared to be linear, and could thus be described for each ripple direction as follows [4, 10]:

φ(ω,Ω)=−2πωτ+2πΩx+χ
( Eq. 9 )

The phase intercepts for downward and upward sweep directions, χ_down_ and χ_up_, contain temporal and spectral components, denoted by θ and ϕ, respectively. This can be written as follows [4, 10]:

$$\chi_{down}=-\theta +\varphi .$$

and

$$\chi_{up}=\theta +\varphi$$
( Eq. 10 )

The separated phase functions from the SVD analysis were used to derive the parameters *τ*, *x*, *θ*, and *φ*. The slope *τ* corresponds to the temporal position (group delay), and the slope *x* corresponds to the spectral position (reflecting BF); these parameters can be determined for both ripple directions. The phase constants *θ* and *φ* are related to asymmetry of the STRF around BF. The parameter *θ* reflects inhibition before and/or after excitation at BF: *θ* < 0 reflects excitation at onset followed by inhibition, and *θ* > 0 reflects inhibition at onset followed by excitation. The parameter *φ* reflects the extent of sideband inhibition above and/or below BF: *φ* < 0 indicates a dominant inhibition below BF, and *φ* > 0 indicates dominant inhibition above BF (see [4], for details).

**Response predictions.** From the STRF, a linear estimate of the temporal response pattern of a cell, R(t), to any sound stimulus can be predicted by time convolution and spectral integration of the STRF with the stimulus spectrogram, S(x, t):

$$R(t)=\int_{F_{\min}}^{F_{\max}}dx\int_{0}^{\infty }d\upsilon .STRF(x,\upsilon ).S(x,t-\upsilon )$$ ( Eq. 11 )

with F_MIN_ and F_MAX_ the minimum and maximum frequencies of the spectral range of the STRF, respectively. The spectrogram is generated using the MATLAB function “specgram” with logarithmic frequency bins. The bin size of the spectrogram S(x, t) in both the spectral and temporal dimension was set according to the bin size of the STRF (dx = 0.25 octaves and dt = 12.5 ms). Because of the limited 2.5 octaves range of the STRFs, we applied a spectral shift to align the STRF maximum with the center of the spectral range, and aligned the stimulus spectrograms accordingly. Convolving the STRF and stimulus spectrogram along the temporal dimension for each spectral bin resulted in a prediction spectrogram$(\int_{0}^{\infty }d\upsilon .STRF(x,\upsilon ).\text{S(}x,t-\upsilon ))$. The prediction spectrogram was then integrated over the spectral domain for each time bin$\text{(}\int_{{\text{F}}_{\text{min}}}^{{\text{F}}_{\text{max}}}\text{dx)}$, which eventually yielded the predicted neural response *(R(t))*. Via this method, we predicted the responses to six animal vocalizations.

## Results

### General properties

We recorded neural activity during ripple presentation from 426 AC neurons (248 in monkey T, 178 in monkey J), which showed at least an acoustically elicited onset or offset response. Further systematic responsiveness of neurons to moving ripples (Ω ≠ 0) and AM complexes (Ω = 0) was quantified by the parameter q (Eq. 4; see Materials and Methods; summarized in Table 1). Forty-one cells (~10%) responded to both moving ripples and AM complexes. Three cells responded only to moving ripples, while 115 cells (27%) responded only to AM complexes. More than half of the neurons (63%, N = 267) demonstrated no significant response to the set of moving ripples, nor to the set of AM complexes, and were therefore excluded from further analysis.

**Table 1:** Number (N) of neurons sensitive to moving ripples (Ω ≠ 0) and AM complexes (Ω = 0) according to their q_25_ and q_50_-values (q_25_>0.387 for Ω ≠ 0 and q_50_>0.376 for Ω = 0).

N = 426 (246)		Ω ≠ 0		
		**responsive**	**non-responsive**	**Total**
Ω = 0	responsive	**41 (39)**	**115 (78)**	156 (117)
	non-responsive	**3 (3)**	267 (126)	270 (129)
	Total	44 (42)	382 (204)	426 (246)

The numbers in parentheses indicate the number of the cells that produced the driven response to pure tone stimuli (see Materials and Methods). Bold numbers indicate the cells that were selected for further analysis (N = 159 for ripples, and N = 120 to both tones and ripples).

Tone responses were also recorded for 246 cells (Table 1; using the same response criteria as in [34]; see Materials and Methods).

**Figure 2:**
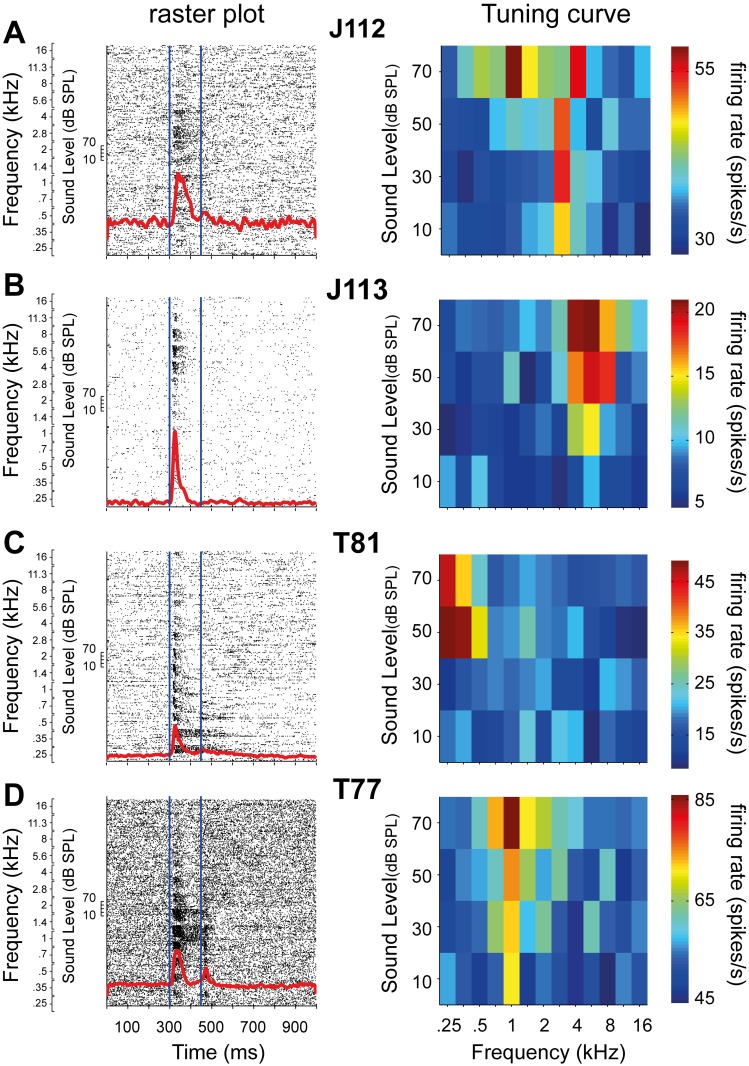
Example tone responses. The raster plot (left) and tuning-curve (right) of four example cells: A. J112, B. J113, C. T81, and D. T77. Note the onset responses in the raster plots. The tone duration (150 ms) is indicated by the blue lines. The responses are sorted first by frequency (0.25–16 kHz), and second by the sound levels (10–70 dB SPL). The tuning curves represent the responses for the various sound level—frequency combinations. The excitatory response bandwidths generally increase by sound level.

**Figure 3:**
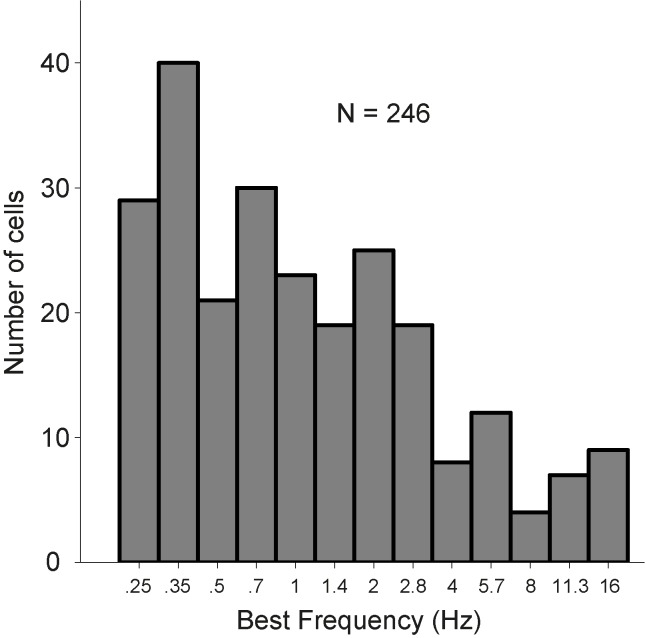
Best frequency (BF) range. From 255 cells responsive to either moving ripples or AM complexes, 246 cells were also responsive to pure tones. The BFs spread across the presented tone stimuli; however, the majority preferred the lower frequencies.

Fig. 2 shows the pure-tone responses and corresponding tuning curves of four example cells. The raster plots (left-hand columns) show each neuron’s spiking activity, which significantly increased above background during tone presentation (from 300–450 ms; blue lines). The four cells showed a clear short-latency onset peak, whereas cell T77 also had a clear offset response (Fig. 2D). The tuning curves (right-hand columns) represent the mean response (averaged over the tone duration) as a function of sound level and frequency. Each of the cells was tuned to a narrow range of frequencies, and their tuning widths typically broadened at higher sound levels. Two cells responded to their preferred frequency at all presented sound levels (10–70 dB SPL; Figs. 2A and D), while the other two only responded at the higher sound levels (Figs. 2B and C). We encountered both monotonic (Figs. 2B and 2D) and non-monotonic (Figs. 2A) rate-level characteristics in our sample of cells. In general, the AC cells in our study showed high sensitivity to pure tones in terms of a low sound level threshold, short response latency, and/or narrow frequency tuning. Best frequencies (BFs) ranged from 0.25 to 16 kHz, with the majority of encountered BFs below 2 kHz (76%; Fig. 3).

**Figure 4:**
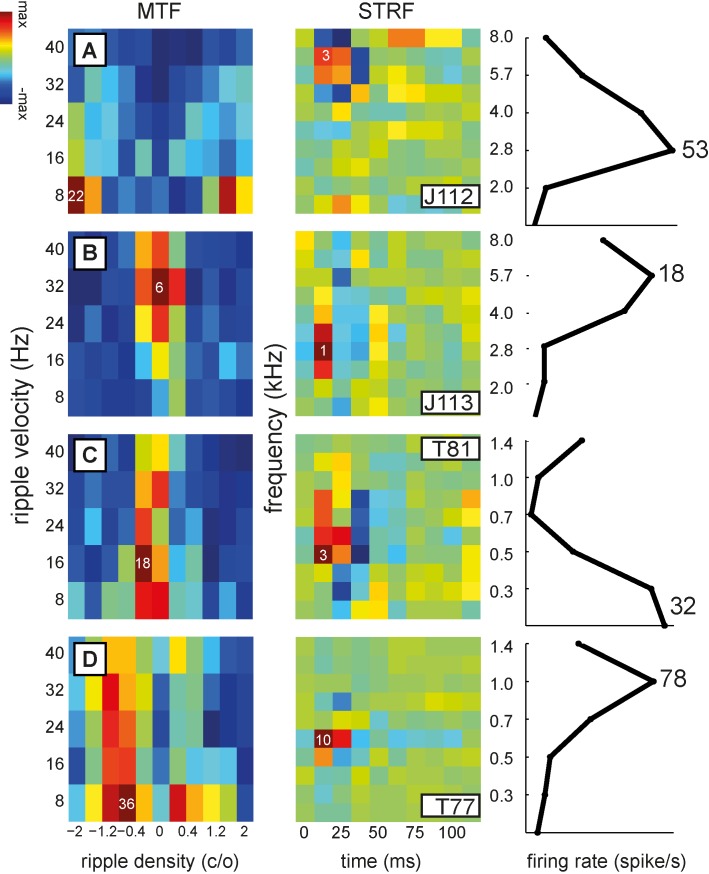
Magnitude transfer functions, corresponding STRFs, and tone responses of the cells shown in Fig. 2. A. J112, example of low best ripple velocity (*ω*_B_ = 8 Hz), and high best ripple density (Ω_B_ = −2 c/o); furthermore it prefers only moving ripples. Note the large difference (1.25 octaves) between BF_tone_ and BF_strf_. B. J113, example of high best ripple velocity (*ω*_B_ = 32 Hz) and low best ripple density (Ω_B_ = 0 c/o). It is responsive to both moving ripples and AM complexes. The difference between BF_tone_ and BF_strf_ is one octave. C. T81, example of asymmetric transfer function with preference for upward direction (Ω<0) with Ω_B_ = −0.4 c/o and *ω*_B_ = 32 Hz. The difference between BF_tone_ and BF_strf_ is 0.5 octaves. D. T77, another example of preference for low velocity moving ripples (*ω*_B_ = 8 Hz; Ω_B_ = −0.8 c/o).

In Fig. 4 we present the magnitude transfer functions and corresponding STRFs for the four example cells shown in Fig. 2. As the spectral range of the STRFs extended to 2.5 octaves (see Materials and Methods), the spectral position of the STRFs is ambiguous. To solve this ambiguity, we used the tone-evoked tuning curves (right-hand column), to determine the appropriate spectral range for each cell (see Material and Methods).

An important feature revealed by the magnitude transfer functions is each cell’s selectivity to a limited range of ripple densities, Ω, and velocities, ω, which strongly varied among the neurons. We encountered neurons that preferred low (Figs. 4B–C) or high ripple densities (Fig. 4A), and neurons that preferred low (Fig. 4A), or high ripple velocities (Fig. 4B). Also, direction selectivity varied, as neurons could strongly prefer either down- or upward moving ripples (Fig. 4C–D, in these examples upward), or responses to both directions were equally strong (Fig. 4A–B). Two of the neurons responded better to moving ripples than to AM complexes (Fig. 4A, D), while the two other neurons responded to both moving ripples and AM complexes (Fig. 4B,C).

The STRFs (Fig. 4, center column) generally showed excitation for a narrow range of frequencies with latencies between 10 and 30 ms, so that a BF_strf_ could easily be obtained (Methods, and see below). Inhibition occurred around the time of or after excitation (Fig. 4A–D). Interestingly, the frequency tuning reflected by the STRF did not agree well with the neuron’s tuning to pure tones. The difference between best frequencies could be as large as 1.25 octaves for cell J112 (Fig. 4A), one octave for cell J113 (Fig. 4B), 0.5 octaves for cell T81 (Fig. 4C), or 0.75 octaves for cell T77 (Fig. 4D). Such a discrepancy suggests a nonlinearity in the response behavior of AC cells (see also below).

**Figure 5:**
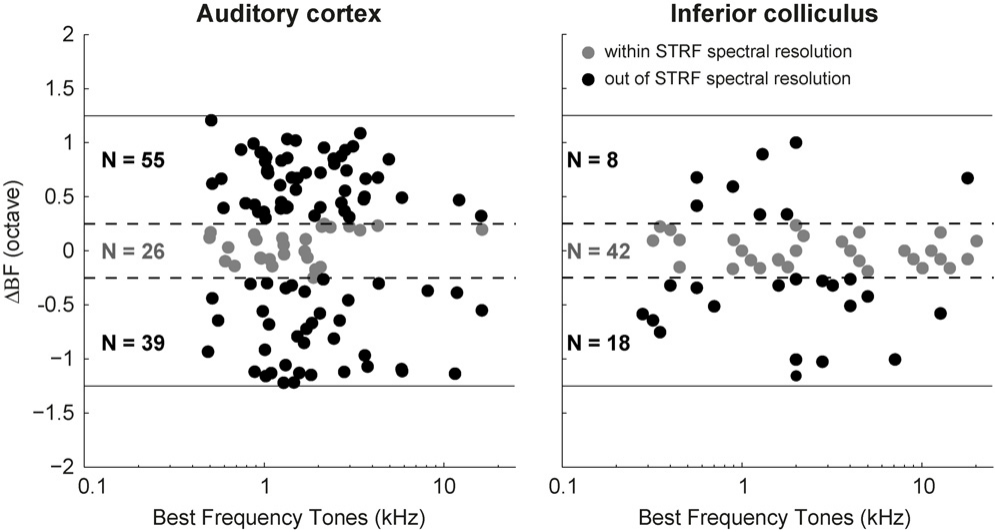
BF_tone_ and BF_strf_ difference (ΔBF). (A) ΔBF obtained for 120 AC neurons that responded both to tones and ripples. (B) ΔBF of 68 neurons recorded from inferior colliculus of monkey (see Fig. 6, Versnel et al., 2009). The grey circles represent the neurons with ΔBF within ±0.25 octaves range. The black circles denote those neurons with −0.25 > ΔBF > 0.25 octaves. The dashed lines frame the ±0.25 octaves borders, and the solid line represents the ±1.25 octaves (the highest possible value for ΔBF). For visual purposes the ΔBF values for AC neurons have been jittered within their corresponding ranges.

To quantify this point in more detail for the population of cells, we plotted the difference between BF_tone_ and BF_strf_ (ΔBF) for all 120 neurons that responded well to both tones and ripples, against BF_tone_ values (Fig. 5A). One should note that the STRF spectral resolution was 0.25 octaves, while the frequency resolution of tone stimuli was 0.5 octaves. We therefore considered the full range of ΔBF between [−0.25, +0.25] octaves as indistinguishable BF values for tones and STRFs. Only 26 of the 120 AC neurons (22%) belonged to this category (grey circles, Fig. 5A).

**Figure 6:**
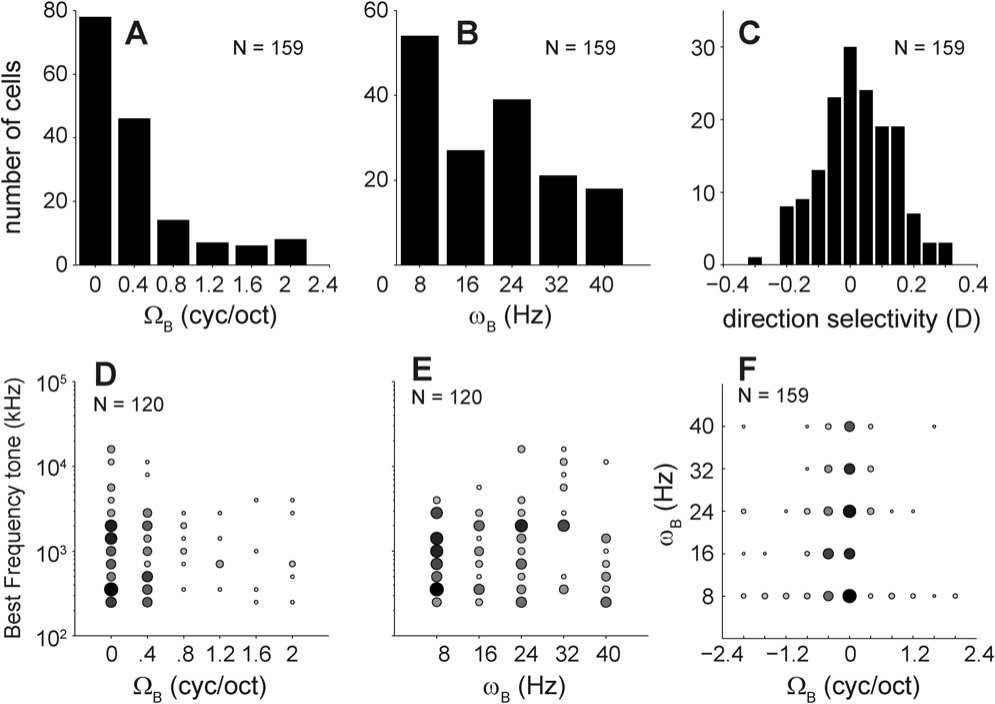
Ripple parameters derived from the magnitude transfer function. Distribution of the A. best ripple density (Ω_B_), B. best ripple velocity (ω_B_), and C. direction selectivity (D). D and E. the relation between best tone frequency versus Ω_B_ and ω_B_, respectively. F. combined distribution of Ω_B_ versus ω_B_, The size of the data points is proportional to the number of cells. For A-C and F the number of cells was 159, and for D and E was 120 for which we had tone responses.

For a direct comparison between AC and IC neurons, we also performed the same analysis for the 68 IC neurons reported in [10]. Here (Fig. 5B), ΔBF of 62% IC cells (42) fell within the ±0.25 range, indicating a much stronger resemblance between BF_tone_ and BF_strf_ and therefore better response linearity for IC neurons.

### Distributions of ripple responses

For all 159 neurons responding to moving ripples and/or AM complexes (83 from monkey T and 76 from monkey J) we determined the best ripple velocity, *ω_B_*, best ripple density, Ω_B_, and the cell’s direction selectivity, *D*, from the modulation transfer function (Figs. 6A-C; see Materials and Methods). The distribution of Ω_B_ was skewed toward low densities, as a large majority of neurons (79%) preferred the two lowest ripple densities (0–0.4 c/o; Fig. 6A). In general, auditory neurons with a low BF_tone_ tend to have broader tuning curves, i.e. low preferred densities (e.g. Kowalski et al., 1995; Versnel et al., 1995 [36, 37]), this low-density preference may simply arise from the low-frequency preference of our recorded AC neurons (Fig. 3). To test for such a potential relation between BF_tone_ and preferred density in our data, we plotted the BF_tone_ versus Ω_B_ for all 120 AC cells for which were driven by tones and ripples. There was no significant correlation (r = −0.07, p = 0.43; Fig. 6D), implying that AC neurons with broad tuning properties (low Ω_B_) do not necessarily follow from a low BF_tone_ preference.

The distribution of best ripple velocities, *ω_B_*, showed a slight preference for low velocities with 34% of AC cells preferring the lowest applied velocity in this study (8 Hz; Fig. 6B). Still a substantial minority (12%) of the neurons preferred the highest velocity tested (40 Hz; Fig. 6B). We observed no correlation between *ω_B_* and BF_tone_ (r = 0.08, p = 0.37; Fig. 6E).

Direction selectivity *D* of the neurons was symmetrically distributed around zero (median D = 0.002), and a majority of neurons (62%) had no preference for upward and downward moving ripples (−0.1 < *D* < 0.1; Fig. 6C). We obtained a strong direction preference (*D* < −0.2 or *D* > 0.2) for 13 neurons (8%). The joint distribution of *ω_B_* and Ω_B_ indicated that ripple selectivity did not cover a wide range of spectral-temporal combinations as few cells were responsive to high *ω_B_* and Ω_B_; furthermore, selectivity to Ω did not correlate with selectivity to *ω*(Fig. 6F). Moreover, direction selectivity was uncorrelated with either *ω_B_* (t_158_ = 23, p < 0.0001) or Ω_B_(t_158_ = 9, p < 0.0001; not shown).

**Figure 7:**
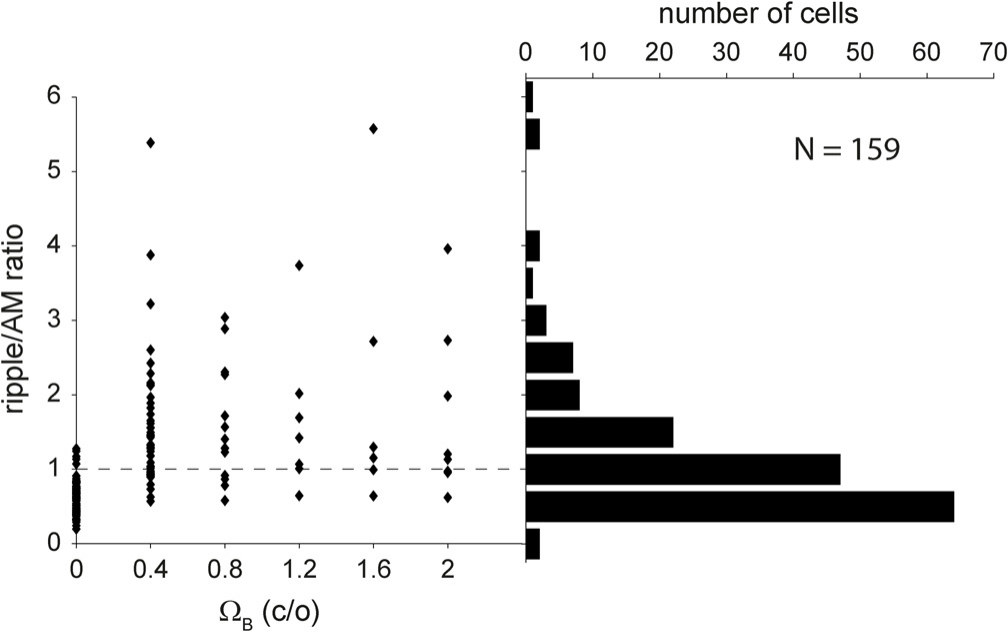
Moving ripple and AM complexes ratio. Ratio of responses to moving ripples and AM complexes as a function of ΩB (*left*), and its distribution (*right*). Bin size, bs, is chosen according to bs = range/√n with range computed over data.

The scatter plot in Fig. 7 (left) shows the ripple/AM ratio, which quantifies the selectivity of the neurons to moving ripples, as a function of best ripple density. As expected, neurons with Ω_B_ = 0 tended to have a ratio below one, while neurons with Ω_B_ > 0 had a ratio above one. Note that exceptions also occurred, as Ω_B_ was obtained at a single velocity, while the ripple/AM ratio was computed across all velocities. The ratio did not depend on Ω_B_>0, which means that, on average, neurons with a low Ω_B_ responded equally strong to AM complexes as neurons with a high Ω_B_. The distribution of the ripple/AM ratio (right-hand side of Fig. 7) was unimodal and skewed towards low ratios, with the peak at a ratio of 0.5 meaning that most neurons (60%) preferred AM complexes rather than moving ripples. Most of the cells preferring moving ripples had their ratio close to one (median = 1.5) implying that they responded nearly as well to spectrally flat AM complexes as to moving ripples (cf. Table 1).

### Spectral-temporal separability

**Figure 8:**
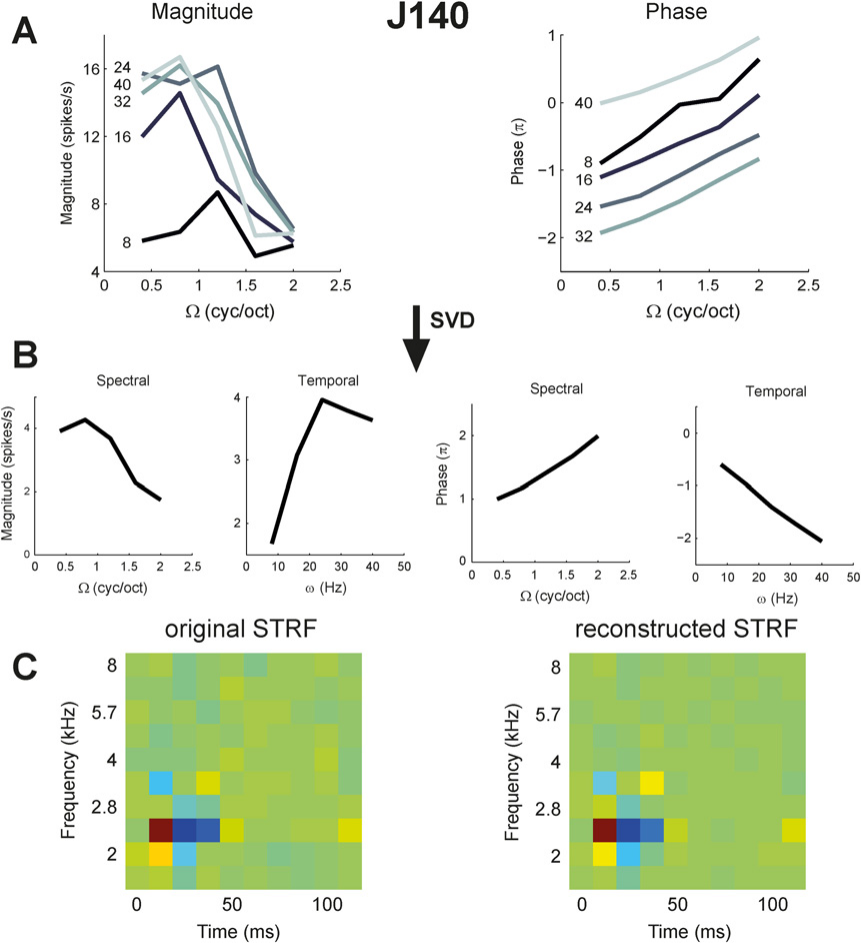
SVD analysis results. SVD analysis of modulation transfer functions (magnitude and phase), shown for downward ripple direction (Ω>0) of an example cell. For this example cell, the eigenvalues (normalized with respect to λ1) are λ1 = 1, λ2 = 0.18, λ3 = 0.1, λ4 = 0.06, λ5 = 0.004, and the total inseparability index α = 0.027. A. Transfer function of downward ripples in Cell J140. B. Separated spectral and temporal transfer functions corresponding to λ1. C. The original STRF (left) and a reconstructed STRF (right) by multiplying separated transfer functions in section B according to Equation 6. The correlation between the STRFs was 0.98.

For a separable transfer function the spectral transfer functions taken at fixed, different velocities (*ω)* should be scaled versions of one another. The same holds for the temporal transfer functions taken at different densities, Ω. We first quantified separability with the SVD method (see Materials and Methods). Fig. 8 shows the result of the SVD analysis on the transfer function of neuron J140 for downward ripple directions (Ω > 0). At velocities 16, 32, and 40 Hz the magnitude curves peaked at ~0.8 c/o, and for velocities 8 and 24 it peaked at ~1.2 c/o. The phase curves were all very similar, running parallel to each other (Fig. 8A). The same property holds for upward ripple directions (not shown). These similarities imply a high degree of separability, which is indeed reflected by a low inseparability index (Eq. 8, Materials and Methods) for this neuron (α = 0.05). The responses can therefore be well described by the mean separated spectral and temporal tuning curves (corresponding to λ_1_), obtained from SVD (Fig. 8B). Indeed, the STRF reconstructed by multiplying the temporal and spectral functions for the magnitude and phase characteristics of both directions and AM complexes (Eq. 7) were very similar to the original STRF (*r* = 0.98) (Fig. 8C).

**Figure 9:**
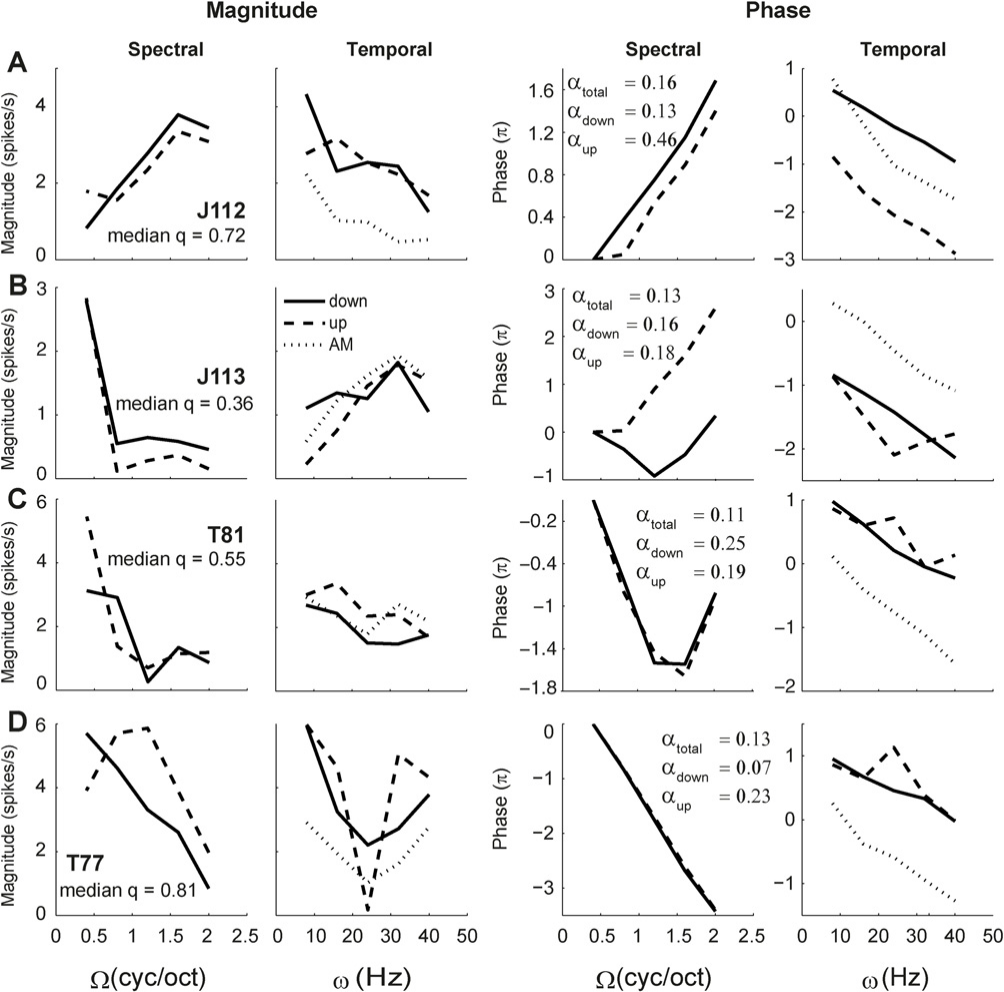
Separated spectral and temporal transfer functions of the neurons shown in Figs. 2 and 4. Magnitude (spikes per seconds) and phase (radians) versus ripple density and ripple velocity. Solid curves represent functions for downward sweep direction (Ω>0), and dashed curves represent functions for upward direction (Ω<0). Dotted curves represent AM transfer functions (Ω = 0). For comparison purposes, the spectral data for Ω<0 are shown along the positive Ω axis. The corresponding magnitude transfer functions are shown in Fig. 4. The total and quadrant inseparability indices (α) are mentioned in the spectral phase plots.

Fig. 9 shows the separated transfer functions (cf. with format Fig. 8B) of the four neurons of Fig. 4. Three of these neurons responded to a sufficiently wide range of ripples to allow for a meaningful SVD analysis (median *q* over total transfer function >0.5; Figs. 9A, C, and D). Neuron J113 responded to only a limited range of ripples, mostly at zero density (q = 0.36; Figs. 4B and 9B). In the quadrant-separated representation of the data, the shape of the transfer functions for the two ripple directions (solid and dashed curves, respectively) appeared quite similar. Note also that the phase functions could typically be well approximated by straight lines (in the range with substantial responses), as predicted by Eq. 8, where only the intercepts of the phase curves could differ for the two directions. The slopes of the phase curves, which reflect the spectral position and group delay (see Eq. 9), were similar (note that slopes for Ω < 0 are shown inverted, for clarity). As all four neurons responded well to AM complexes, their AM transfer functions are plotted as dotted lines in the temporal graphs for comparison. Note that also the AM curves were similar to the separated ripple transfer functions, differing only in magnitude.

**Figure 10:**
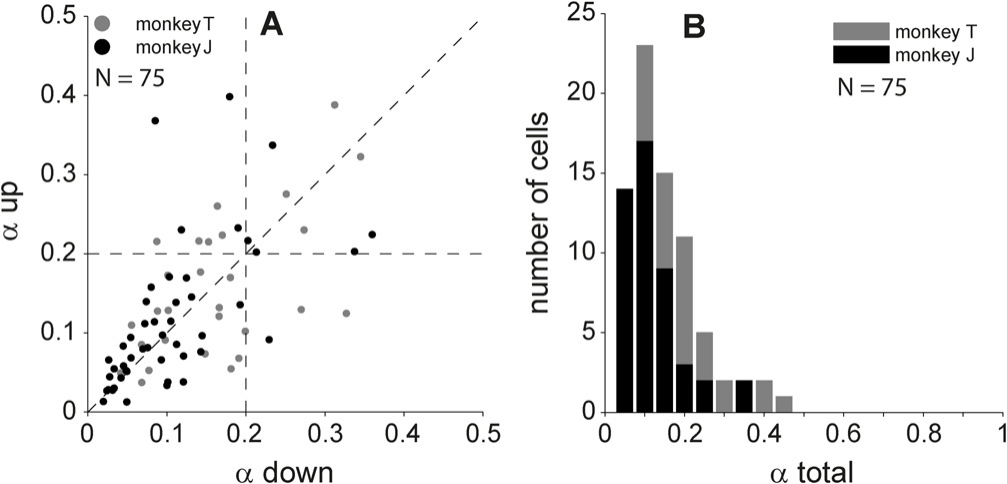
Inseparability indices. A. Distribution of quadrant separabilties, α_down_ vs. α_up_. B. Distribution of total separability index (α_total_). The number of cells, for which the q > 0.4, was 75.

We performed the SVD analysis (Eq. 8) for all cells in our population that had good temporal following over a wide range of ripples (median *q* > 0.4; *N* = 75 cells). Fig. 10A shows that for a large majority of selected neurons the transfer functions in both directions were separable (α_up and down_< 0.2 for 72% of the neurons). For 12 neurons (16%) good separability was obtained for one direction only (α_up or down_< 0.2). The direction for which the lowest α was obtained typically coincided with the preferred direction, D. A statistical simulation indicated that the probability of finding a value α < 0.3 for a random 5×5 transfer function is <0.01. Using α = 0.3 as a selection criterion for (in)separability, we found that six of the single-direction transfer functions were inseparable, and that only two neurons had inseparable transfer functions for both directions. The distributions per animal were very similar (gray and black dots and bars in Fig. 10 represent each monkey). In conclusion, AC neurons show a significant degree of spectral–temporal separability for a single direction, a feature that has become known as quadrant separability [4, 10].

The value of α for the complete transfer function normally exceeds the lowest index obtained separately for the two directions. Assuming quadrant separability, we also performed a statistical simulation on complete 11×5 transfer matrices that consisted of three components: two different quadrant-separable 5×5 matrices representing transfer functions to both directions, and a 1×5 matrix representing the AM noise transfer function. When the three components differed randomly, we obtained a probability <0.01 of finding a value α < 0.3. Using α = 0.3 as a criterion, we found that only 7% (5 cells) of the complete transfer functions were inseparable (Fig. 9B). The inseparability indices for these transfer functions were confined to an intermediate range of values (0.3 < α < 0.5).

**Figure 11:**
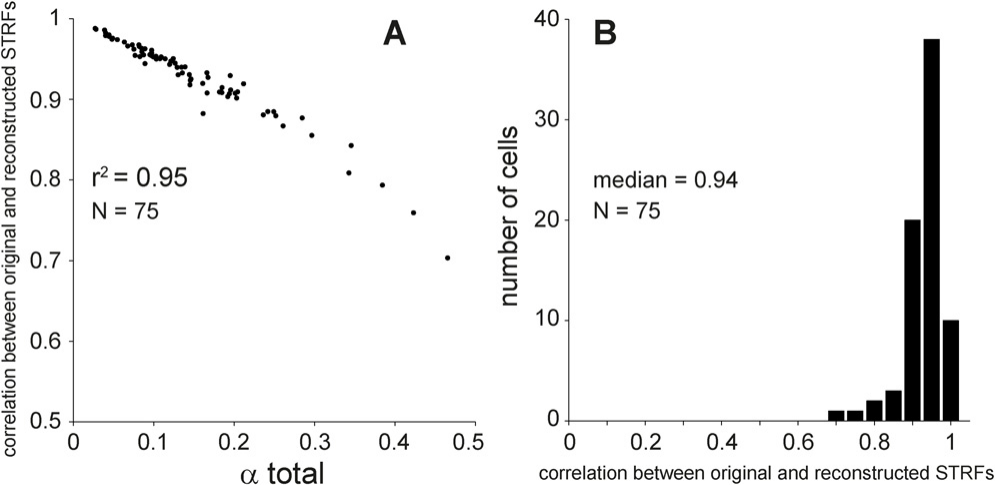
Correlation between original and reconstructed STRFs (*ρ*). A. *ρ* as a function of total inseparability index (α_total_). B. distribution of *ρ*.

Because the data indicated quadrant separability for the AC neurons that responded well to moving ripples (see above), the full transfer function *T*_sep_(*ω*, Ω) can in principle be obtained by determining only the temporal and spectral transfer functions, *F_B_*(*ω*) and *G_B_*(Ω), and subsequently applying Eq. 6: *T*_sep_(*ω*, Ω) = *F_B_*(*ω*) × *G_B_*(Ω) (cf. Fig. 8C). To test whether this alternative approach would yield a good STRF estimate, temporal and spectral slices *F*(*ω*, Ω = Ω_*B*_) and *G*(Ω, *ω* = *ω_B_*) were taken from the recorded transfer function, *T*(*ω*, Ω), at their optimal velocity *ω_B_* and density Ω_B_. The correlation ρ between *T* (*ω*, Ω) and *T*_sep_(*ω*, Ω) was computed as a measure of separability, with ρ = 1 indicating perfect separability (cf. α = 0). We verified that ρ was indeed significantly related to the inseparability index α (*r* ^2^ = 0.95; Fig. 11A). The median ρ was 0.94, confirming the high degree of quadrant separability and validity of this approach to estimate the STRF of a separable cell (Fig. 11A). The correlation coefficients ρ were also estimated separately for the amplitude and phase of the transfer function, resulting in a median ρ of 0.88 for amplitude and a median ρ of 0.67 for phase. This indicated that the separability of amplitude was higher than for phase.

### Phase functions

**Figure 12:**
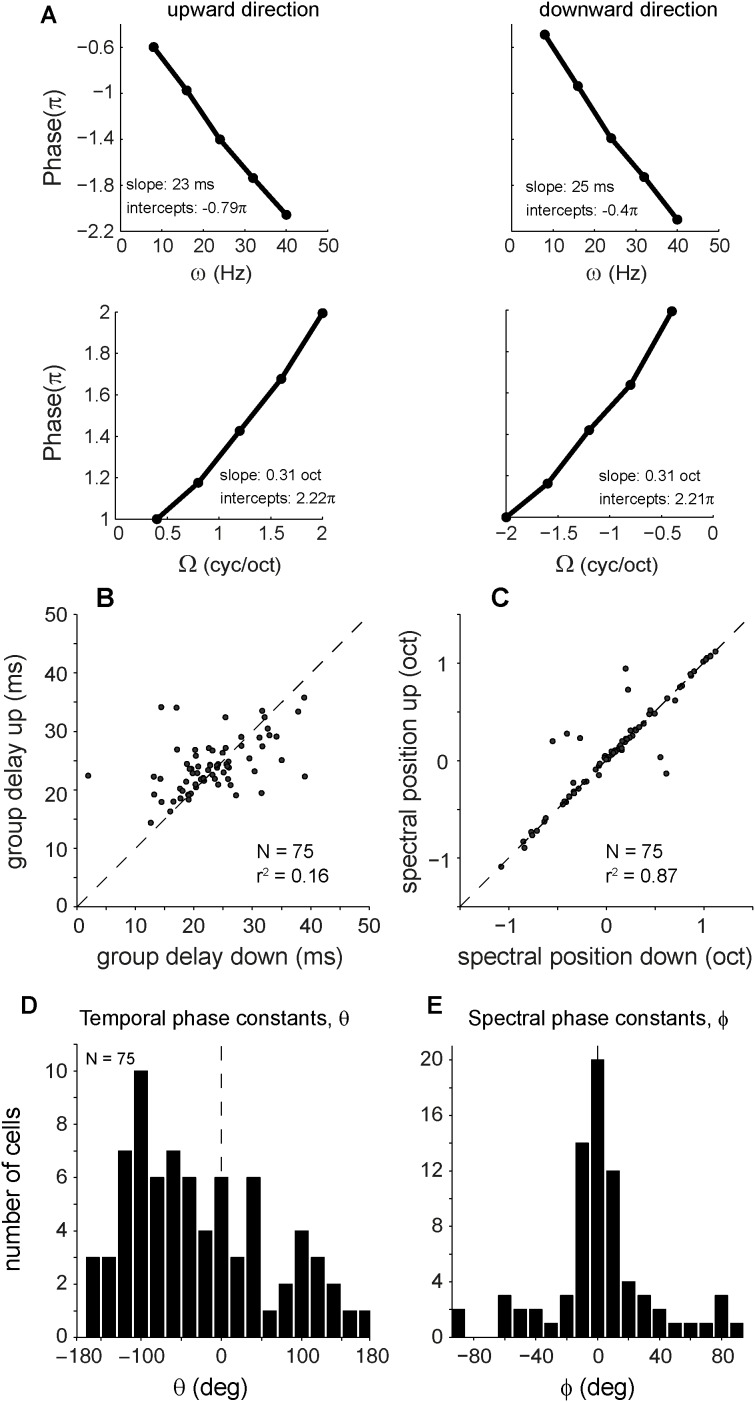
Phase properties. A. Phase functions of neuron shown in Fig. 8 (J140). Phase, derived from SVD (see Fig. 8B), plotted as a function of velocity and density. For both directions, slopes correspond to temporal position (group delay) and spectral position (BF), see Equation 8. B. Temporal positions, or group delays, for upward versus those for downward sweep direction. C. Spectral positions for upward versus those for downward sweep direction. The spectral positions are between −1.25 and 1.25 octaves. When the difference between the two positions is > 1.25 octaves, a 2.5 octaves correction is made. D. Distribution of temporal phase constant θ (−180°<θ<−180°). F. Distribution of spectral phase constant φ (−90°<φ<90°).

The phase functions, such as shown in Figs. 8 and 9, could be approximated by straight lines. This feature, which results from phase locking to the ripple envelope, is further quantified in Fig. 12A by fitting linear regression lines through the temporal and spectral phase data for upward (left) and downward (right) ripples (same neuron as in Fig. 8). The slopes and intercepts of the regression lines yielded meaningful parameters (see Eq. 9): the slopes can be associated to group delay and spectral position, whereas the spectral-temporal parameters derived from the intercepts (see Eq. 10) correspond to STRF asymmetries in excitatory and inhibitory sidebands in the spectral and temporal domains.

Fig. 12B shows that the group delays obtained for the population of 75 cells fell in a range of 15–40 ms. The group delays for upward and downward ripple directions agreed within 3 ms for a majority of neurons (72%). The group delays were longer than the onset latencies to tone stimuli by approximately 5 ms, which was significant (t_67_ = −4.7, *p* < 0.001). The group delays did not correlate with the tone-evoked latencies (*r* ^2^ = 0.02; *p* = 0.23), and the latency differences did not correlate with direction selectivity *D* either (*r* ^2^ < 0.001; *p* > 0.9). The spectral positions found for the two directions had almost identical values for a large majority of neurons (Fig. 12*C*). Spectral position differences also did not correlate with preferred ripple direction (*r* ^2^ = 0.004; *p* = 0.56).

Fig. 12*D* shows that the temporal phase constant θ varied between −180° and 0° for most neurons (64%), which implies an onset excitation at BF_STRF_ (see Materials and Methods). We obtained a positive θ for a substantial minority of neurons (36%). This feature hints at an inhibitory onset response for the STRF. The spectral phase constant *φ* (Fig. 12*E*) was almost normally distributed around 0, which means that dominant inhibition above (*φ* > 0) and below (*φ*< 0) a cell’s BF was found in a similar number of neurons. A large majority of neurons has a *φ* near 0 indicating symmetry of sideband inhibition above and below BF.

**Figure 13:**
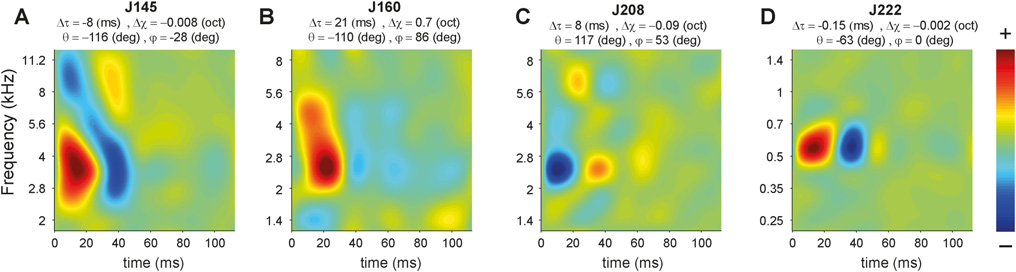
The effect of spectral and temporal phase values on STRF. A-D. For each cell different phase values are mentioned above its corresponding STRF. Δt represents the difference between group delay (ms) of downward and upward directions responses (see Fig. 12B). Δχ denotes the difference of spectral position of downward and upward ripple responses in octaves (see also Fig. 12C). The θ value represents excitation (θ<0) or inhibition (θ>0) on onset response (see Fig. 12D), and φ value demonstrates spectral asymmetry around BF_strf_ (see also Fig. 12E). A. an onset cell (θ<0) with inhibition merely above the BF_strf_ (φ< 0). B. an onset cell (θ<0) with inhibition below the BF_strf_ (φ>0). The Δχ value implies 0.7 octaves difference between BF position for downward and upward ripple responses, which is obvious from the broad spectral excitation pattern. C. an offset cell (θ>0) with excitation only below the inhibition spot (φ>0). D. An onset AC cell responding equally to both upward and downward ripples (small Δt and Δχ values), which has also symmetrical small inhibition above and below the BF_strf_.

To illustrate how the spectral and temporal phase values influence the STRF, Fig. 13 shows four example AC cells with different phase values. Δt represents the difference between group delay (ms) of downward and upward directions responses (see also Fig. 12B). Δχ denotes the difference of spectral position (BF_strf_) of downward and upward ripple responses in octaves (see also Fig. 12C).

The cells in Figs. 13A, B, and D are examples of a negative temporal phase (θ < 0), which indicates excitation that is followed by an inhibitory response. If θ >0, the neuron exhibits an inhibition that is followed by an excitatory response (Fig. 13C, see also Fig. 12D). Values for φ unequal to zero imply a spectral sideband asymmetry in the STRF. For an onset cell, like in Figs. 13A and B, the inhibitory sideband is asymmetric, while for an offset cell such as in Fig. 13C, the excitatory sideband is asymmetric. Negative values for φ indicate an asymmetry only above the BF_strf_(e.g. Fig. 13A), whereas positive values correspond to an asymmetry only below the BF_strf_, (Figs. 13B and C). Fig. 13D shows an example cell (J222) without asymmetric inhibitory sidebands (φ = 0).

Cell J160 (Fig. 13B) has a relatively large Δχ value. As a result, the spectral positions for downward and upward directions (see Fig. 12C) are 0.7 octaves apart. This is reflected by a spectrally broad excitation, which ranges from about 2 kHz up to 5.6 kHz. Note that this cell also had a large difference between the response latencies for both ripple directions (Δt = 21 ms). In contrast, cell J222 (Fig. 13D) responded virtually identical to the downward and upward ripple directions as indicated by the small values for Δt and Δχ.

### Linear predictions

**Figure 14:**
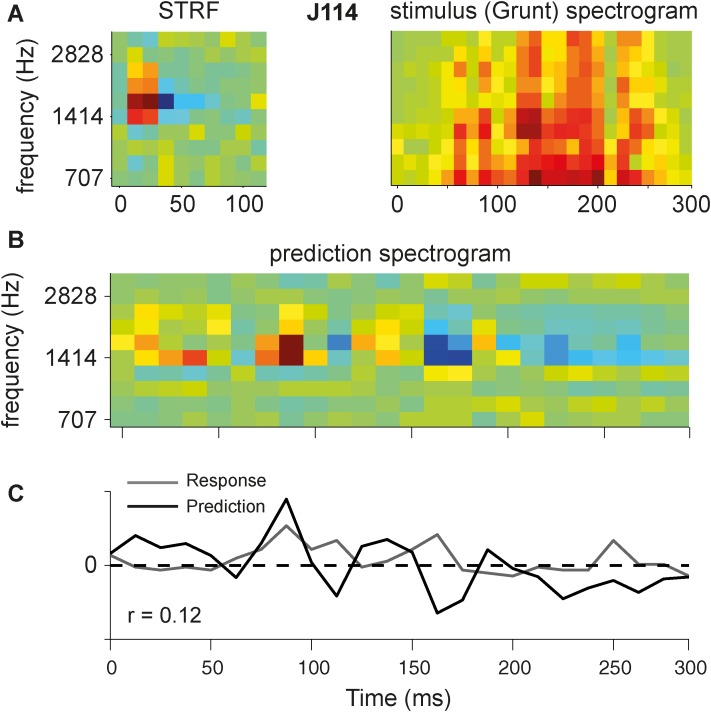
Prediction response of an example cell (J114). A. STRF and spectrogram of the stimulus (monkey vocalization, grunt). The STRF is spectrally centered around its BF. The spectrogram was taken at the same frequency range and the same spectral-temporal resolution. B. Prediction spectrogram yielded by convolution along the temporal dimension of STRF and stimulus spectrogram. C. Predicted response (black) obtained by summation along the spectral dimension of predicted spectrogram. The gray curve represents the actual response for comparison. The average spontaneous spike rate (here, 58 spikes/s) is subtracted from the actual response. The amplitude of the predicted response is scaled to the actual response.

For 75 cells with good temporal following to a wide range of ripples (median q > 0.4), we also analyzed the responses to six different natural vocalizations. We predicted the responses to the vocalizations by applying the linear STRF model of Eq. 11 (details in Materials and Methods). The result of this analysis for one of the neurons (J114) is illustrated by the black curve in Fig. 14C for the monkey grunt sound. The actual average response (gray curve) is shown for comparison. Note that the predicted response was not rectified; negative values thus indicate a predicted response inhibition, while positive values can be interpreted as the firing rate of the cell. Although the largest peak of the actual response was successfully predicted, the linear model failed to make a decent prediction for the rest of the trial, as evidenced by the low correlation coefficient between the two curves (r = 0.12).

**Figure 15:**
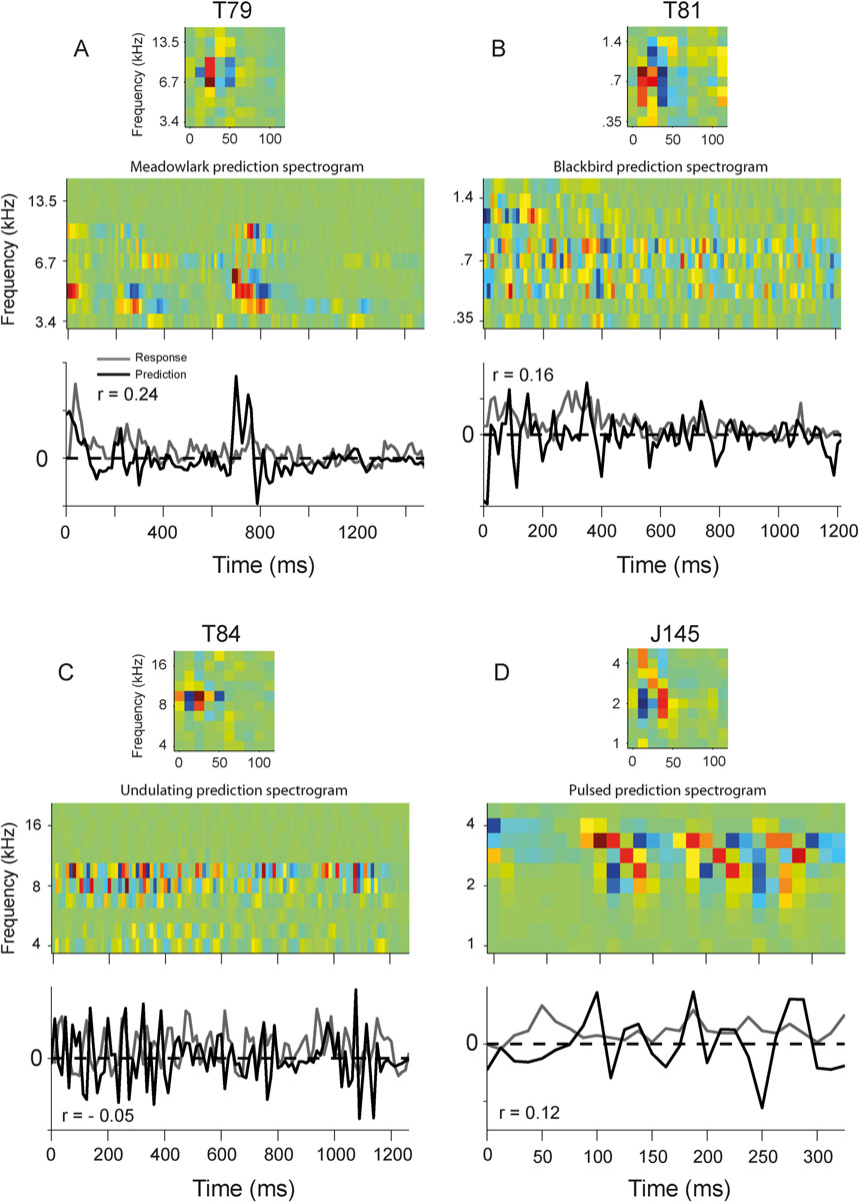
Prediction of response to four different vocalizations compared to actual response for four AC cells. A. Cell T79 with high BF (6.7 kHz) responding to bird call (Meadowlark). B. Cell T81 with low BF (0.8 kHz) and a broad excitation, which followed by inhibition, responding to Blackbird call. C. T84, an inhibitory onset cell with high BF (10 kHz) responding to undulating vocalization. D. J145, another inhibitory onset cell with broad inhibition-excitation and medium BF (2kHz). The predicted (black) and actual (gray) responses are shown for each cell. In general the prediction was poor.

Fig. 15 shows the prediction for four of the other vocalizations presented to different neurons of the two monkeys. Although most acoustic energy of these vocalizations overlapped considerably with each neuron’s STRF, the linear predictions were still poor.

**Figure 16:**
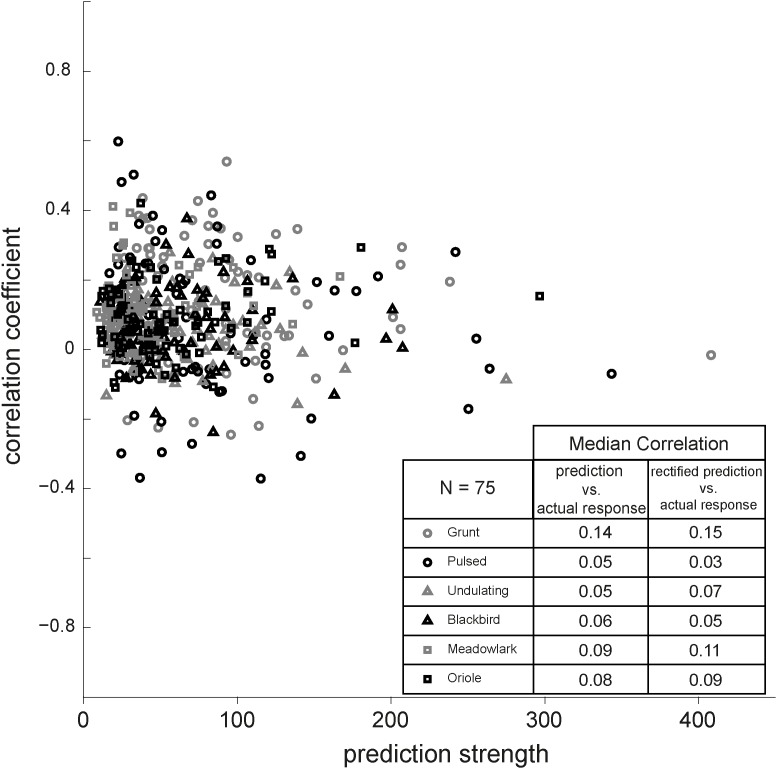
Predictability and prediction strength. Correlation between actual and predicted responses to 6 vocalizations as function of the prediction strength for 75 AC cells. The table shows the median correlation of recorded response versus predicted and rectified predicted responses, respectively. Rectification did not improve the prediction.

To summarize the overall performance of the linear predictive power of the STRF for all vocalizations across the 75 neurons, Fig. 16 shows the response correlations as a function of each neuron’s prediction strength. We defined the latter as the root-mean-square of the predicted response amplitude for each vocalization, to avoid trivially low correlations for stimuli for which the expected response would be close to zero. In our previous study of monkey IC neurons, the highest linear response correlations were typically obtained for the strongest responses [10]. In contrast, however, we found no such relationship for the population of AC cells. In general, the correlations between actual and predicted responses were low for each of the six vocalizations, as shown in the table. The best predictions were obtained for the monkey grunt sound (r = 0.14, on average), although they were still very low. Rectification of the predicted responses, by setting the negative values to zero, had no significant influence on these results.

## Discussion

Our study allows for a direct comparison of the spectral-temporal tuning properties of AC cells with those obtained from IC cells in awake macaque monkeys, as they were tested under the same experimental protocols and for the same sound stimuli [10]. Our results indicate that both structures share a wide diversity of selective responses to ripple densities and velocities (Figs. 4 and 6), and that both demonstrate quadrant and full spectral-temporal separability in the majority of recorded neurons (Fig. 10). However, we also observed marked differences between the IC and AC cells. First, while the correspondence between best frequencies for tones and ripples remained within 0.25 octaves for most IC cells, it was quite poor, with often more than half an octave difference, for the population of AC cells (Fig. 5). Second, the linear prediction for AC responses to the set of natural stimuli on the basis of the neuron’s STRF was typically poor (Figs. 14–16), whereas for IC neurons the prediction was fair (correlations 0.4–0.6) for stimuli yielding high firing rates.

### General characteristics

**Spectral tuning.** The AC population showed a predominant preference for low ripple densities (Ω_B_ ≤ 0.4 c/o; Fig. 6A). This result is similar to monkey IC [10], and to data obtained from thalamus and A1 in anesthetized animals [2, 14, 38–41]. This suggests that ripple-density selectivity might be faithfully transmitted from IC to AC, and that anesthesia may have little influence on spectral tuning characteristics. A similar preference for low ripple densities was also reported for human auditory cortex [42], and for human psychophysics [43]. This prominent preference for low ripple densities might be confounded by the high percentage (76%) of recorded AC cells with a low BF_tone_ (<2 kHz), considering that low BF_tone_ cells typically have broader tuning characteristics (e.g. Kowalski et al., 1995; Versnel et al., 1995 [36, 37]). However, the absence of a correlation between BF_tone_ and Ω_B_ and the low Ω_B_ values for high-BF_tone_ neurons observed in this non-human primate study, indicate that neurons across monkey AC have low Ω_B_ (Fig. 6D).

**Temporal tuning.** Previous studies have suggested that the range of preferred ripple velocities decreases from IC to cortex [40, 41]. In line with these studies, the peak at low ω_B_ in our study was similar to that of monkey IC (ω_B_ = 8 Hz for 34% of AC cells, vs. 31% for IC cells; [10]), but preference for the two highest measured velocities has decreased substantially from IC to AC (ω_B_ = 32–40 Hz in 36% of IC cells vs. 24% of AC cells; Fig. 6A; [2, 10, 14, 40]). Also, the joint distribution of optimal velocities and densities for the AC population did not cover the entire 2D spectral-temporal space as for IC neurons [10]. For example, cells tuned to higher ω_B_ (> 24 Hz), tended to be associated with a smaller range of best densities than cells tuned to low ω_B_ (Fig. 6F). This suggests that AC cells can process sound features over a range that is spectrally narrower than IC cells [10].

**Phase functions.** The phase functions of the spectral-temporal transfer function were approximately linear in both quadrants (Fig. 12A), which was also reported for ferret A1 neurons [2, 4] and for monkey IC [10]. These straight-line relationships indicate good phase following of the ripple envelopes. The regression coefficients derived from these straight-line phase relations are informative descriptors of the STRF: the slope τ of the temporal phase function determines the group delay (BF_strf_ latency), whereas the slope χ of the spectral phase function determines its spectral position (BF_strf_). Like reported for IC neurons, these parameters were symmetrically distributed for both upward and downward ripple directions for AC cells as well (Fig. 12B, C). However, unlike IC neurons, there was no significant relation between the up-down differences of direction selectivities (D) and the up-down group-delay differences [10].

The spectral (φ) and temporal (θ) components of the intercepts of the straight-line phase functions refer to STRF asymmetries in excitatory and inhibitory bands around the cell’s BF. Like for ferret A1 [2, 4] and monkey IC [10], we found similar distributions for these intercepts. The majority of AC neurons have an onset excitation at their BFs (θ < 0; Fig. 12D). Inhibitory side bands above (φ > 0) and below (φ < 0) the BF_strf_ were found for the same fractions of AC neurons (Fig. 12E).

### Separability and direction selectivity

In anesthetized preparations a substantial proportion of A1 cells were characterized by inseparable STRFs (ferret, [4, 44]; cat, [40]; mouse, [5]). Most often, however, inseparability was due to an asymmetry between ripple directions (Ω>0 vs. Ω<0), as the transfer functions for inseparable STRFs were typically quadrant separable. Versnel et al. [10] reported that the majority of monkey IC neurons (>70%) had fully separable STRFs, and IC cells with inseparable STRFs were typically quadrant separable. We here found that STRFs of AC cells were even more separable than IC neurons, since 93% of the cells were both fully separable and quadrant separable. Our results may be quite surprising since direction selectivity is typically found to increase from IC to cortex (rat, [45]) and separability is found less often in awake than in anaesthetized conditions (ferret, [44]). We suggest that species differences underlie the apparent differences in (in)separability between previous studies and our results from awake monkey. The extent of separability may arise from the species-specific relevance of environmental sounds (e.g., vocalizations in bat have with strong spectrotemporal direction dominance leading to high inseparability of STRFs [17]).

### Linear response predictions

As a simple measure for a potential nonlinearity in AC responsiveness we took the difference between the best frequencies found for pure tones (BF_tone_) and for the STRF derived from broadband ripples (BF_strf_). Our results revealed poor similarity between the two BFs (Fig. 5). Previous studies demonstrated a strong correspondence between the BFs in A1 cells of anesthetized ferrets [2, 38, 39], which could suggest that anesthesia might linearize potentially nonlinear response behavior of AC neurons. Recordings taken from IC showed a much better agreement between BF_tone_ and BF_strf_ in awake bats [17], as well as in awake monkeys (Fig. 5B, [10]). This might indicate that the contribution of nonlinear processing to the neural responses increases along the ascending auditory pathway.

Note that the BF_tone_ is based on a spike-rate average response (see Materials and Methods). Conversely, the STRF relates to the synchronization with the ripple envelope modulations. This could lead to different BFs for pure tone and modulated sound stimuli. Previous studies have demonstrated a gradual transformation from temporal encoding (synchronized) at lower auditory system levels, to a rate coding mechanism at higher levels [46]. One would expect increased resemblance, rather than an increased difference between the BF_tone_ and BF_strf_ for AC cells relative to IC as both BFs in AC would be based on non-synchronized firing rates. However, this would not happen, as it has been reported by other studies that both AC and IC neurons of awake monkeys are well capable of phase locking above the modulation frequencies range (8:8:40 Hz) used for the AC and IC neurons [47]. Thus, although for both AC and IC neurons the STRF was based on synchronized responses, this led to a large discrepancy between the two measures of BF for AC cells, when compared to the IC cells [10].

In addition, we observed a general failure to faithfully predict responses to the set of vocalizations on the basis of the STRF (Figs. 14–16). Previous studies, applying the STRF method, demonstrated that most AC neurons responded linearly to broadband sounds in rats [13], to ripples in anesthetized ferrets [11, 39, 48], and to virtual acoustic space stimuli in anesthetized ferrets and cats [12, 49]. This could suggest that cortical responses may be more linear in anesthetized preparations than in awake animals. If so, the apparent discrepancy between results may be attributed to the state of alertness of the animal [16, 50]. Note that other studies have shown that differences in an animal’s alertness do not systematically change the shape of the STRF [25, 51], but rather seem to affect the response strength [9].

When the neural response behavior is nonlinear, the shape of a neuron’s STRF, and hence its potential usefulness to predict responses to other stimuli, may critically depend on the stimuli used to extract the STRF, as well as on their resemblance with test stimuli used to compute the linear prediction, or response approximation [52–56]. Indeed, broadband sustained stimuli, such as the spectral-temporal ripples used in this study, led to a different estimate of the STRF for cortical neurons than narrow-band stimuli, like natural vocalizations [48]. In line with this, some studies have reported that the linear STRF prediction of AC cells improves with the similarity between test stimuli and the sounds used to derive the STRFs (in anesthetized ferrets [39]; in anesthetized zebra finches [52]). We therefore suspect that part of the failure to predict AC responses to the set of vocalizations in Figs. 14–16 may be explained by such nonlinear aspects, as the STRFs were derived from responses to ripples, which differed substantially from the natural vocalizations. Although it is feasible that better predictive power to natural sounds could be obtained if STRFs are derived from similar natural sounds, a quantitative comparison with earlier IC data requires the use of the same data analysis procedures and stimulus sets [10].

Single-unit recordings from the IC of awake bats [17] and monkeys [10] showed a fair linear predictability of the responses. Those results, however, also indicated that IC responses could not be fully explained by a linear kernel such as the STRF. This could imply an increased contribution from nonlinear processing along the ascending auditory pathway from IC to AC, which is in line with the observations of Atencio et al. [57]. The data of Yeshurun et al. [58, 59] showing that natural sound responses were better predicted by medial geniculate body (MGB) neurons than by cortical neurons, indicate that nonlinearities could arise at the level of the AC. This is further corroborated by studies reporting multiplicative encoding of different sound features in ferret AC [60], and multiplicative interactions between bottom-up (acoustic) and top-down (task-related) signals in single units of monkey AC [25, 26].

Our findings add to a growing body of evidence for a hierarchical increase of auditory processing complexity along the auditory ascending pathway. Through a detailed comparison with data earlier obtained from the monkey IC [10], we found that the spectral-temporal acoustics across the IC and AC populations, as described by the STRF and responses to pure tones, are very similar (Table 2). Yet, prominent differences between the two structures have become apparent as well: the tuning characteristics for tones, ripples and natural stimuli are strongly related at the level of single IC neurons, where the STRF provides a reasonable model to predict the responses to the different classes of stimuli, while this correspondence is nearly lost for AC neurons. Whether these differences are due to intrinsic neural nonlinearities such as short-term plasticity or rapid synaptic depression [61], increased trial-to-trial response variability [62], additional top-down cognitive signals [25, 26], or to a combination of these factors, needs to be explored in future studies.

**Table 2:** Summary of different spectral and temporal characteristics of AC and IC cells.

	**Ω_B_ (c/o) distribution**	**ω_B_(Hz) distribution**	**Direction selectivity (D)**	**BF_tone_ and BF_strf_ aggreement**	**Spectral and temporal separability**	**Response predictability (linearity)**
**Auditory cortex**	broad (median±std = 0.4 ± 0.55)	skewed to low values (median±std = 16 ± 11)	no strong preference (median±std = 0.02 ± 0.12)	poor (22%)	high (93%)	very poor (r<0.1)
**Inferior colliculus**	broad (median±std = 0.4 ± 0.54)	skewed to low values (median±std = 16 ± 12)	no strong preference (median±std = 0.01 ± 0.13)	robust (62%)	high (71%)	fair (r~0.45)
